# Safety and efficacy of fingolimod in Iranian patients with relapsing-remitting multiple sclerosis: An open-label study

**DOI:** 10.22088/cjim.12.3.263

**Published:** 2021-04

**Authors:** Rozita Doosti, Abdorreza Naser Moghadasi, Amir Reza Azimi, Shahrokh Karbalai Saleh, Masoud Etemadifar, Vahid Shaygannejad, Fereshteh Ashtari, Mohammad Hossein Harirchian, Seyed Bahaadin Siroos, Hormoz Ayramloo, Nastaran Majdinasab, Seyyed Mohammad Masood Hojjati, Nabiollah Asghari, Seyed Mohammad Baghbanian, Hamed Cheraghmakani, Mahmoud Abedini, Behnaz Sedighi, Negar Mohseni Abbas abadi, Maedeh Ghasemitabar, Sara Talebianpour, Tohid Babayi Daylari, Vahid Dana, Neda Ghaleh noie, Mohammad Ali Sahraian

**Affiliations:** 1 Multiple Sclerosis Research Center, Neuroscience Institute, Tehran University of Medical Sciences, Tehran, Iran; 2 Department of Cardiology, Sina Hospital, Tehran University of Medical Sciences, Tehran, Iran; 3 Department of Functional Neurosurgery, Medical School, Isfahan University of Medical Sciences, Isfahan, Iran; 4 Isfahan Neuroscience Research Center, Alzahra Research Institute, Isfahan University of Medical Sciences, Isfahan, Iran; 5 Isfahan Neuroscience Research Center, Isfahan University of Medical Sciences, Kashani MS center, Isfahan, Iran; 6 Iranian Center of Neurological Research, Neuroscience Institute, Tehran University of Medical Sciences, Imam Khomeini Hospital, Tehran, Iran; 7 Department of Neurology, Imam Reza Hospital, Tabriz University of Medical Sciences, Tabriz, Iran; 8 Department of Neurology, Golestan Hospital, Ahwaz University of Medical Sciences, Iran; 9 Department of Neurology, Babol University of Medical Sciences, Babol, Iran; 10 Faculty of Medicine, Semnan University of Medical Sciences, Semnan, Iran; 11 Department of Neurology, Booalisina Hospital, Mazandaran University of Medical Sciences, Sari, Iran; 12 Department of Neurology, Mazandaran University of Medical Sciences, Sari, Iran; 13 Neurology Research Center, Kerman University of Medical Science, Kerman, Iran; 14 R&D Department, Osve Pharmaceutical Co., Tehran, Iran; 15 Quality Assurance Department, Osve Pharmaceutical Co., Tehran, Iran

**Keywords:** Fingolimod, Multiple Sclerosis, Safety, Efficacy, EDSS

## Abstract

**Background:**

Fingolimod was the first oral therapy approved for treating relapsing-remitting multiple sclerosis (RRMS) in 2010. This open-label study evaluated the safety and efficacy of fingolide^R^, 0.5 mg in Iranian MS patients during one-year follow-up.

**Methods:**

A multicenter, open-label, longitudinal was designed to evaluate the safety and efficacy of fingolide^R^, 0.5 mg over a one-year follow-up period across 11 centers. The patients were visited by their neurologists every two months to evaluate possible adverse events and clinical disease activity considered by recording Kurtzke’s Expanded Disability Status Scale (EDSS).

**Results:**

A total of 252 patients with the mean treatment duration of 343±45.70 days were. 20 patients experienced adverse events (AEs) and serious adverse events (SAEs) such as resistant urinary tract infection (UTI), premature atrial contraction (PAC), skin allergic reaction, macular edema, chicken pox, zona, panic attacks, and exacerbations associated with steroids treatment, all of which led to Fingolide^R^ discontinuation. The mean EDSS decreased from (2.15±1.29, 95%CI: 1.99to2.32) at baseline to (1.85±1.22, 95%CI: 1.68to2.02) at 12th month (final visit) while a p-value revealed significant differences comparing baseline and final EDSS (p<0.001). Mean annualized relapse rate (ARR) of the patients in one year prior to the study was (0.006±0.016, 95%CI: 0.004to0.008) which changed to (0.005±0.016, 95%CI: 0.003to0.007) at the end of the study period. Patients with a 12-month period of fingolide^R^ treatment experienced sustained ARR and disease progression (p<0.001).

**Conclusion:**

The obtained findings suggest that the administration of Fingolide^R^, 0.5 mg (Fingolimod, Osvahpharma, Tehran, Iran) is safe and efficient for Iranian MS patients.

Multiple Sclerosis (MS), as the most common autoimmune disease involving the central nervous system, predominantly affects young adults (1-3). MS is the first cause of non-traumatic neurologic disability and the second cause of disability after trauma in young population. Environmental and genetic factors seem to be the etiology of MS (4). New findings have confirmed an increase in the incidence and prevalence of MS in the Iranian population (5). Over the last two decades, various types of treatments such as interferon-β preparations, glatiramer acetate (GA), mitoxantrone, natalizumab, teriflunomide, and dimethyl fumarate have been approved to prevent the disability and exacerbation of MS (6, 7). Gilenya® (Fingolimod, Novartis, Basel, Switzerland) as the first oral disease-modifying therapy was approved for relapsing-remitting MS in 2010.

The mentioned drug binds to sphingosine-1-phosphate (S1P) receptors that are expressed on lymphocytes to prevent lymphocyte egress from lymph nodes and a result of which prevents the auto-aggressive lymphocytes from crossing the blood-brain barrier (8). FREEDOMS, TRANSFORMS and FREEDOMS II are the phase III trials in RRMS patients and have indicated the efficacy of fingolimod in reducing the annualized relapse rate (ARR) (0.18 vs. 0.40 (9), 0.16 vs. 0.33, and 0.21 vs. 0.40, respectively) (10, 11). Cardiovascular adverse events (AEs) such as bradycardia, heart blocks, and various types of arrhythmias lead to a 6-hour cardiac monitoring of the first-dose administration of fingolide (12-14), and the monitoring time may be extended under special conditions (15-17). Increased incidence of varicella-zoster virus (VZV) infections (18) and severe HSV encephalitis have been reported in some immunized patients (19, 20). Observed macular edema with or without visual symptoms in few patients in the mentioned trials has led to the recommendation of the ophthalmological evaluations before and 3-4 months after the treatment initiation (21, 22). Few cases complicated by abnormal fetal growth, especially in the first trimester, as well as cases with spontaneous abortions are advised to use effective contraception during the treatment with fingolimod (23, 24). Furthermore, patients planning a pregnancy should stop treatment with fingolimod at least two months before conception. Rare cases of melanoma (25), progressive multifocal leukoencephalopathy (PML) (26), acute lymphoblastic leukemia (ALL) (27), disseminated cryptococcosis (28), lymphomatoid papulosis (29), and reversible cerebral vasoconstriction syndrome (RCVS) (30) have been reported in the literature following its marketing approval. Several studies conducted in local and multinational contexts have demonstrated the efficacy and safety of fingolimod across different populations. The obtained real-world data re-emphasize the findings presented in previous clinical trials (31-35). Recent findings have confirmed the effectiveness of fingolimod in western Iranian RRMS patients (36). Another clinical trial have shown the significant superiority of Fingolide in comparison with high-dose interferon beta-1a in Iran (37). The present study addresses the first administration of Fingolide^R^ that was distributed in Iranian market in 2013. According to Iran’s law, non-biologic drugs do require clinical trials to gain approval and can enter the market after conduction of *in vitro* bioequivalence studies. Hence, the present study was designed to examine the safety and efficacy of generic Fingolide^R^, 0.5 mg (Fingolimod, osvahpharma, Tehran, Iran) in Iranian RRMS patients.

## Methods

**Study design: **The present multicenter, open-label, longitudinal study was designed to enroll relapsing patients from 11 centers from January 2013 to June 2015 and evaluate the disease course before and after the intervention during a one-year follow-up study. The involved 11 centers were located in 10 cities across Iran. The study was conducted in accordance with the ICH Harmonized Tripartite Guidelines for Good Clinical Practice and the Declaration of Helsinki (38, 39).

Patients aged 18-65 years were diagnosed with RRMS based on McDonald criteria (2010), had the Kurtzke’s Expanded Disability Status Scale (EDSS) score ranging between 0-5.5, had at least one documented relapse experience during one year prior to the initiation of the study while using first-line disease modifying drugs (DMD_s_), presented intolerability of or severe side effects associated with interferon beta, reported no relapse during the last month prior to the initiation of the study, gained negative result of pregnancy test in case of being a childbearing woman, and indicated positive varicella-zoster antibody in serum were included in the study.

In addition, patients who were diagnosed with RRMS accompanied by any chronic diseases other than MS such as diabetes mellitus, uncontrolled hypertension, malignancy, active pulmonary or cardiac disease, myocardial infarction, or cerebrovascular accident over six months before enrollment, had any evidence of infectious diseases such as active bacterial, fungal, or viral infection, had macular edema at onset, and were treated with immunoglobulin or monoclonal antibodies or immunosuppressive drugs such as azathioprine and methotrexate over six months before screening were excluded from the study. Furthermore, patients who were previously treated with cyclophosphamide or mitoxantrone, were diagnosed with secondary or primary progressive MS, were pregnant at the onset of the study or had any decisions for being pregnant during the study period, and had abnormal ECG (any block, QT_c _interval more than 440 ms on screening ECG) were not included in the study. 

Written informed consent was obtained from all eligible patients at the screening visit before conducting any study-related procedures. The neurologist evaluated patients’ conditions based on basic data such as demographic information, a past medical history, past MS medications, expanded disability status scale (EDSS) score, etc. Laboratory tests performed prior to enrollment included CBC/diff, LFT_S_ (AST, ALT), urine analysis (U/A), varicella-zoster antibody-titer, tuberculin skin test, and β-HCG for women. Cardiology and ophthalmology consultations were performed for all patients prior to the initiation of the study. Furthermore, the ophthalmological evaluation was performed prior to and three months after the initiation of the therapy as well as every six months thereafter to monitor any changes with a special focus on macular edema (ME). All patients received oral fingolide^R^ 0.5 mg (Fingolimod, osvahpharma, Tehran, Iran) and underwent first-dose monitoring. Then, they used oral fingolide^R ^0.5 mg per day and were visited by their neurologists every two months to evaluate the possible adverse events of fingolide and the clinical activity of disease (as relapse) in a one-year follow-up study. Kurtzke’s EDSS was recorded at each visit following the same method during the whole observation period. First-dose monitoring was extended to 24 hours in case of any changes in cardiac rhythm, with the exception of asymptomatic bradycardia.

To prevent any heterogeneity in the obtained data from the centers, uniform definitions of the variables were considered by the examining physicians. Physical examination and disability status were assessed by Kurtzke’s Expanded Disability Status Scale (EDSS) (40). Relapse was defined as the appearance or reappearance of one or more significant neurological abnormalities that persist for at least 24 hours and are immediately preceded by a period of relatively stable or improved disease condition for at least 30 days (10). Normal fluctuations in a patient’s MS symptoms did not themselves constitute a relapse. A relapse was confirmed by the examining physicians when the patient’s symptoms were accompanied by objective changes on the neurological examination and an increase of at least 0.5 EDSS or an increase of 1 point in 2 FS or 2 points in one of the FS (excluding bowel and bladder) (36).

Safety assessments included records of adverse events that were obtained from the patient history, physical examination, and laboratory assessments. Treatment-emergent adverse events would be followed till resolution. An adverse event(AE)was considered as any undesirable experiences affecting the patient’s health and occurred during the study regardless of being related to the study treatments or not. A serious adverse event (SAE) or reaction was considered as any untoward medical occurrence, which resulted in persistent or significant disability or incapacity, death, inpatient hospitalization, or prolongation of existing hospitalization. Life-threatening outcome or congenital anomaly and birth defect were investigated as SAE. Follow-up was required until the AE or SEA and its sequel resolve or stabilize at a level acceptable to the investigator. Definitions of AEs and SAEs were all considered based on previous reports in the literatures (9-11). Efficacy assessments included recordings of relapses during the study period and Kurtzke’s EDSS score for each patient (40).

The present study was approved by the Ethics Committee of Tehran University of Medical Sciences (approval number 91-01-85-17187-59311) and was also submitted to Iranian Registry of Clinical trial (IRCT) (approval number: IRCT201112267419N4). All protocols as well as study design were approved by the local ethics committee.

**Statistical analysis: **Statistical analysis was performed using SPSS Version 22. Demographic and baseline characteristics such as sex, age, disease duration, baseline and final EDSS scores, and relapse rate were analyzed. Data was presented as means ± SD for continuous variables and as frequency (percentage) for categorical variables. Frequency of AEs and SAE_s _was calculated at 95% confidence interval considering the type and number of events per percentile. The significance level was set at 0.05 level. To assess the possible heterogeneity of the EDSS score among the centers, a likelihood ratio test (LR Test) was used in R software Version 3.5.2. For this purpose, two models were implemented as follows: the centers were ignored and included in model 1 and 2, respectively. Finally, two models were compared using LR test. All centers had access to the site to fill the data by the same rater during the study period. The extracted data were monitored to review the study progress at regular intervals. To allow the use of the information provided by this research, all the extracted data was assessed and analyzed by one statistician.

It was estimated that the enrollment of 256 individuals would be required considering the alpha level to be 0.05 (z=1.96), , the prevalence of any adverse events to be 60%, the maximum acceptable error (d) equal to be 6%, and the findings of the study be conducted by Laura Ordonez-Boschetti (31). 

## Results

**Figure1:**
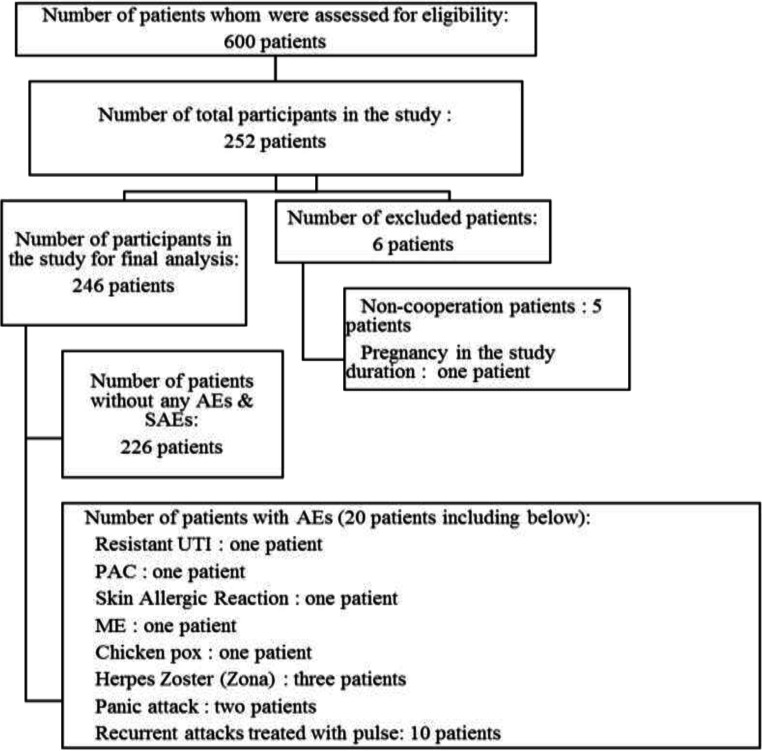
Enrollment and follow-up of patients in this study

A total of 252 patients from 11 centers across all regions of Iran were enrolled in this study. The baseline characteristic information including age, duration of MS diagnosis, number of prior MS treatments, baseline and final EDSS, and duration of fingolide treatment is reported in table 1. Furthermore, information regarding the enrollment and follow-up of patients in this study is presented in figure 1. 

In the present study, the mean age of the patients was 31.62±7.18 (mean±SD) years. Specifically, 182 (77.4 %), 10 (4.3%), 2 (0.85 %), and 2 (0.85%) patients had a history of using interferons, GA, Gilenya, and Tysabri, respectively. In addition, 39 (16.6 %) patients had no history of treatment before the study enrollment. The main concomitant medications that the patients received before the enrollment were vitamin-D_3_ (n=92, 37.4 %), omega-3 (n=54, 22 %), calcium-D (n=31, 12.6 %), vitamin-E (n=27, 11 %), vitamin-B_1_ (n=27, 11 %), gabapentin (n=21, 8.9 %), and multivitamin (n=20, 8.1 %). A total of 246 (97.62%) patients completed their participation in the study, while 6 (2.38%) patients were excluded from the study (one patient because of pregnancy and five patients lost to follow-up).

**Adverse Events (AEs) and Serious Adverse Events (SAEs): **Details of AEs and SAEs are indicated in table 2. Totally 250 (99.21%) patients in this study presented at least one AE during one-year follow-up. The most commonly registered AEs were related to events such as UTI, dyspnea, blurred vision, headache, raised LFT_s_, alopecia, depression, GI upset, hypermenorrhea, and fatigue. Zona and chicken pox were observed only in three (0.97%) and one (0.32%) patients, respectively and led to discontinuation of fingolide. Furthermore, during one-year follow-up, only one 26-year-old male patient (0.32%) with no history of cardiological disorders showed PAC as the cardiac emergency about 6 months after starting fingolide. The mentioned finding led to the discontinuation of fingolide treatment. Only one confirmed case of unilateral macular edema (ME) (0.32%) in a 26-year-old female patient was reported in the present study. ME was presented in this patient with blurred vision, decreased visual acuity, and eye pain. It was diagnosed within 3 months after the initiation of fingolide treatment, and visual aquity returned to the baseline 6-8 weeks after the discontinuation of fingolide. Seizure happened in five (1.62%) patients with no previous history. Changes in menstrual pattern, which affected patients’ sexual life, was another event observed among female patients. Hypermenorrhea occurred with the highest frequency (1.62%) among female patients. Notably, no cases of death or malignancy were reported.

The most common reason leading to the treatment discontinuation was recurrent attacks treated with pulse therapy in 10 (47.63%) patients. The other reasons leading to the treatment discontinuation are presented in table 4. According to the findings presented in table 4, only one (4.76%) patient had pregnancy experience during the study period. The mentioned patient was excluded from the study. During the study follow-up period, the mentioned patient reported spontaneous abortion at 8^th^ gestational age. Although all AEs were mild to moderate, all led to the treatment discontinuation.

**First-Dose Monitoring: **Detailed information of first-dose monitoring events in the present study is presented in table 3. Most of the patients (n=219, 89.02%) were discharged after routine monitoring, which lasted six hours. Self-limited headache resolved with simple analgesic was recorded in 3.66% of the patients during the first-dose monitoring. Self-limited asymptomatic bradycardia resolved within routine monitoring was recorded in seven (2.85%) patients. Despite the mentioned finding, three (1.22%) patients presented symptomatic bradycardia, which was self-limited and required no treatment.

First-degree AV-block was developed in two (0.82%) patients and resolved within 24 hours after the first-dose administration of fingolide. The mentioned observation led to the discontinuation of fingolide administration. Three (1.23%) patients had shown symptoms such as chest pain, headache, dyspnea, etc. within routine monitoring and required treatment and C.C.U admission. All of the mentioned patients were discharged following 6-hour monitoring after the second-dose fingolide. In line with the protocol, none of the patients had indicated prolonged QTc interval or second- or higher-degree AVB_s_ at final ECG. There were significant differences between the patients with and without complication in the first-dose monitoring (p<0.001) in term of the cardiac events. The mean rates of 112.26±9.89 and 70.88±11.35 were recorded for systolic and diastolic blood pressure before receiving the first-dose of fingolide. Furthermore, a transient decrease in blood pressure started 30 minutes after receiving fingolide at the first-dose monitoring and became more significant over time. Thirty minutes after the administration, the mean systolic and diastolic blood pressure was 110.95±9.34 mm Hg and 70.03±11.32 mm Hg, respectively. The lowest level of blood pressure was observed 4 hours after the initiation of the monitoring and resolved within the last 2 hours of monitoring. Four hours after the initiation of the monitoring, the mean systolic and diastolic blood pressure was 106.55±9.96 mm Hg and 65.8±9.93 mm Hg, respectively. Moreover, the final recorded vital signs indicated 107.18±9.58 mm Hg and 66.11±9.71 mm Hg for systolic and diastolic blood pressure, respectively.

**Figure2:**
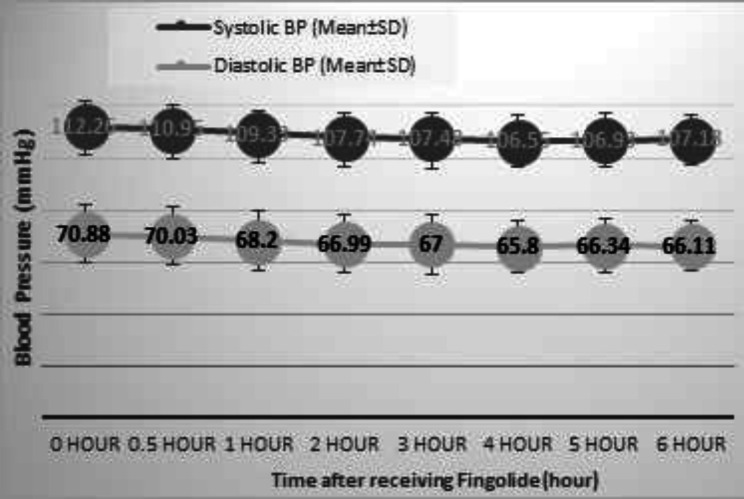
Blood Pressure changes per time after receiving Fingolide

Blood pressure changes over the time after receiving fingolide are presented in figure 2. A comparison between systolic blood pressure before receiving the first-dose of fingolide and after six hours of monitoring had shown significant differences over the time (p<0.001). Furthermore, this comparison for diastolic blood pressure before receiving the first-dose of fingolide and after six hours of monitoring was significant (p<0.001).

**Figure3:**
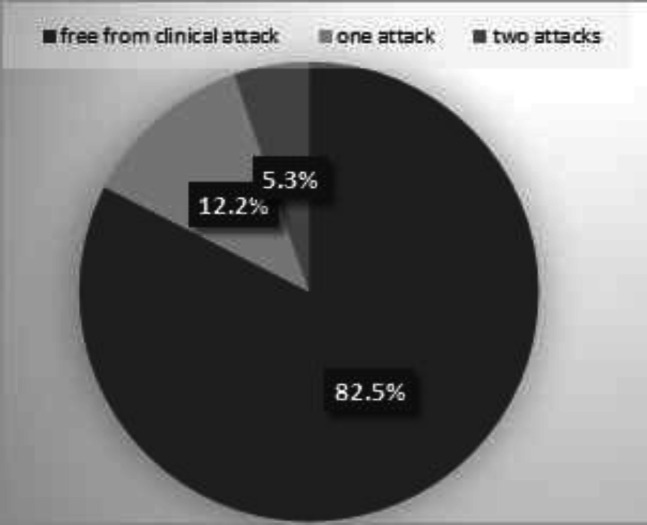
Attack number during one-year follow-up in this study

**Efficacy: **During the study period, the mean EDSS decreased from 2.15±1.29 at baseline to 1.84±1.22 at the final visit over the 12^th^ month; while the p-value revealed significant differences comparing baseline and final EDSS (p<0.001). To check the possible heterogeneity of the outcomes among the centers, two models were implemented. The centers were ignored in model 1, whereas they were included in model 2. Finally, two models were compared using LR test. The results of LR test suggested that there was no significant difference between two models (df=1, chi square=1.35, P=0.245); therefore, the heterogeneity of the outcomes among the centers was rejected. Mean annualized relapse rate (ARR) of the patients over one year prior to the study was 0.006±0.016 (95%CI: 0.004to0.008) and changed to 0.005±0.016 (95%CI: 0.003to0.007) at the end of the study period. Patients with a 12-month period of fingolide treatment experienced sustained ARR and disease progression (p<0.001). Furthermore, 203 (82.5%) patients had no clinical attacks. In addition, 30 (12.2%) and 13 (5.3%) patients had one and two attacks over the study duration, respectively. The attack number over the study duration is presented in figure 3. The finding comparing the patients with and without attacks was significant (p<0.001). 

**Table 1:** Baseline characteristic information of patients in this study

Variable	Total (Mean ± SD)	Min	Max	95%CI
**Age (years)**	31.62±7.18	18	52	30.71to32.53
**Duration of MS diagnosis(months)**	72.31±49.68	1	217	65.86to78.77
**Number of prior MS treatments**	1.64±1.21	0	6	1.49to1.80
**Baseline EDSS**	2.15±1.30	0	5.5	1.99to2.32
**Final EDSS**	1.85±1.22	0	5.5	1.68to2.02
**ARR of one year prior to the study**	0.006±0.016	0.00	0.14	0.004to0.008
**ARR at the end of the study period**	0.005±0.016	0.00	0.14	0.003to0.007
**Duration of Fingolide treatment(days)**	343.80±45.70	178	423	337.64to349.96

SD: Standard Deviation, MS: Multiple Sclerosis, EDSS: Expanded Disability Status Scale 95%CI: (95% Confidence Interval), ARR: Annualized Relapse Rate

**Table 2:** AEs and SAEs of Fingolimod during one-year follow-up in this study

Outcome	N (%)	95%CI	Outcome	N (%)	95%CI
AEs:			AEs:		
Infections:			Skin reactions:		
**UTI**	33(13.5%)	0.094to0.183	Alopecia	12(4.9%)	0.025to0.084
**URI**	9(3.6%)	0.017to0.068	Skin lesions	4(1.6%)	0.004to0.041
**Flulike **	4(1.6%)	0.004-0.041	Itching	3(1.2%)	0.003to0.035
**Herpes Zoster (Zona) **	3(1.2%)	0.003to0.035	Hair loss	2(0.8%)	0.000to0.029
**Chicken pox **	1(0.4%)	0.000to0.022	Psychologic:		
**Cardiologic:**			Depression	14(5.7%)	0.031to0.094
**Dyspnea **	9(3.7%)	0.017to0.068	Anxiety	4(1.6%)	0.004to0.041
**Hypertension crisis **	4(1.6%)	0.004to0.041	Insomnia	2(0.8%)	0.000to0.029
**Dizziness **	3(1.2%)	0.003to0.035	Panic attack	2(0.8%)	0.000to0.029
**Chest pain **	2(0.8%)	0.000to0.029	Amnesia	1(0.4%)	0.000to0.022
**PAC**	1(0.4%)	0.000to0.022	Gastrointestinal:		
**Ophthalmologic:**			GI upset	4(1.6%)	0.004to0.041
**Blurred vision **	6(2.4%)	0.009to0.052	Constipation	2(0.8%)	0.000to0.029
**Diplopia **	4(1.6%)	0.004to0.041	Abdominal pain	2(0.8%)	0.000to0.029
**Ophthalmic Allergic Reaction **	1(0.4%)	0.000to0.022	Weight gain	2(0.8%)	0.000to0.029
**M.E **	1(0.4%)	0.000to0.022	Weight loss	1(0.4%)	0.000to0.022
**Neurologic:**			Nausea	1(0.4%)	0.000to0.022
**Headache **	37(15%)	0.108to0.201	Increased appetite	1(0.4%)	0.000to0.022
**Limb pain **	13(5.3%)	0.028to0.089	Anorexia	1(0.4%)	0.000to0.022
**Paresthesia **	7(2.8%)	0.012to0.058	Fecal incontinency	1(0.4%)	0.000to0.022
**Spasticity **	6(2.4%)	0.009to0.052	Sexual:		
**Seizure**	5(2%)	0.007to0.047	Hypermenorrhea	5(2%)	0.007to0.047
**Asthenia **	4(1.6%)	0.004to0.041	Nontospecified AUB	4(1.6%)	0.004to0.041
**Tremor **	2(0.8%)	0.000to0.029	Vaginal discharge	3(1.2%)	0.003to0.035
**Ataxia **	2(0.8%)	0.000to0.029	Oligomenurreha	2(0.8%)	0.000to0.029
**Limb weakness **	2(0.8%)	0.000to0.029	Vaginal spotting	2(0.8%)	0.000to0.029
**Restless leg **	1(0.4%)	0.000to0.022	libido decrease	2(0.8%)	0.000to0.029
**Hematologic:**			General:		
**Raising in LFT**_s _	34(13.8%)	0.098to0.188	Fatigue	15(6.1%)	0.035to0.099
**Lymphopenia **	12(4.9%)	0.025to0.084	Puffiness	3(1.2%)	0.003to0.035
**Leukopenia **	5(2%)	0.007to0.047	Epistaxis	1(0.4%)	0.000to0.022
**SAEs:**					
**Pregnancy **	1(0.4%)	0.000to0.022			

AEs: Adverse Events, SAEs: Serious Adverse Events, 95%CI: (95% Confidence Interval), UTI: Urinary Tract Infection , URI: Upper Respiratory Tract Infection, PAC: Premature Atrial Contractions, ME: Macular Edema, LFTS: Liver Functional Tests, GI: Gastrointestinal, AUB: Abnormal Uterine Bleeding

**Table 3:** First-Dose Monitoring of Fingolimod in this study

Outcome	N (%)	95%CI
**Discharged:**	219(89.0%)	0.844to0.926
**Discharged after 6hour monitoring without any problem**	219(89.0%)	0.844to0.926
**Others selftolimited symptoms revealed without treatment :**	18(7.3%)	0.044to0.113
**headache**	9(3.7%)	0.017to0.068
**Asymptomatic bradycardia without treatment ** **(HR less than 45pbm or HR decrease more than 80% of baseline)**	7(2.8%)	0.012to0.058
**Transient diplopia**	1(0.4%)	0.000to0.022
**Weakness & lethargy**	1(0.4%)	0.000to0.022
**Extended monitoring time for more 3hour:**	3(1.2%)	0.003to0.035
**Symptomatic bradycardia & abdominal pain **	2(0.8%)	0.001to0.029
**Symptomatic bradycardia & headache **	1(0.4%)	0.000to0.022
**Extended monitoring time for more 24hour:**	2(0.8%)	0.001to0.029
**First degree AVB** **in final ECG & Symptomatic bradycardia**	1(0.4%)	0.000to0.022
**First degree AVB** **in final ECG without any symptom**	1(0.4%)	0.000to0.022
**CCU admission:**	3(1.2%)	0.003to0.035
**Chest pain & headache**	1(0.4%)	0.000to0.022
**Dyspnea**	1(0.4%)	0.000to0.022
**Symptomatic bradycardia **	1(0.4%)	0.000to0.022
**Exclusion from trial:**	1(0.4%)	0.000to0.022
**Panic attack leading to exclusion from trial**	1(0.4%)	0.000to0.022

95%CI: (95% Confidence Interval), HR: Heart Rate, AVB: Atrioventricular Block, ECG: Electrocardiography, CCU: Coronary Care Unit

**Table 4:** Reasons for discontinuation of Fingolimod in this study

Reasons	N (%)
**Recurrent attacks treated with pulse leading to discontinuation of Fingolimod**	10(47.63%)
**AE**_s_ **:**	
**Zona**	3(14.29%)
**Panic attack**	2(9.52%)
**Resistant UTI **	1(4.76%)
**PAC**	1(4.76%)
**Skin Allergic Reaction**	1(4.76%)
**Macular edema**	1(4.76%)
**Chicken pox**	1(4.76%)
**SAE**_s_ **:**	
**Pregnancy **	1(4.76%)

AEs: Adverse Events,

SAEs: Serious Adverse Events,

UTI: Urinary Tract Infection

## Discussion

Evidence from the present study demonstrated the safety and efficacy profile of generic fingolide in Iranian RRMS population. The mean age of patients in this study (31.62±7.18) was less than that of FREEDOMS, FREEDOMSII, and TRANSFORMS phase III trials (36.6±8.8, 40.6±8.4, and 36.7±8.8 years, respectively). Note that the present study in comparison with FREEDOMS, FREEDOMSII, and TRANSFORMS involved a limited number of participants (252 vs. 425, 358, and 431, respectively), a shorter duration of MS diagnosis (72.31±49.68 months vs. 8.0±6.6, 10.4±8.0, and 7.5±6.2 years, respectively), and lower EDSS (2.15±1.29 vs. 2.3±1.3, 2.4±1.3, and 2.24±1.33, respectively) (9-11). UTI was the most commonly observed infection among the patients in the present study (10.71%). The mentioned finding was not consistent with those of FREEDOMS, FREEDOMSII, and TRANSFORMS trials (8%, 15%, and 6.1%, respectively). The most commonly observed infection in the mentioned trails was URI (49.9%, 52%, and 7.2%, respectively). The presented finding was in contrast with those of the present study (2.92%) (9-11).

Frequencies of hypertension and headache in the present study were 1.3% and 12.01%, respectively, which were less than those of the previous studies (6.1%, 9%, and 3.7% for hypertension and 25.2%, 23%, and 23.1% for headache, respectively) (9-11). Increase in LFTs (11.04%) in the present study seemed to be similar to the findings presented in previous trials (15.8%, 8%, and 6.5%, respectively). In addition, the frequency of lymphopenia (3.9%) in the present study was higher than that of the previous studies (3.5%, 8%, and 0.2%, respectively) (9-11). The mentioned findings represented the mechanism of the pharmacological effects of fingolide. The present trail reported no case of death, which is consistent with the findings of previous studies. Although, no case of malignancy (0%) was reported in the present study, previous trials presented different findings in this regard (0.9%, 4%, and 1.9%, respectively) (9-11). Despite the longer time of follow-up in the previous trials (24 months in both study) as compared with the current study (12 months), this finding may be due to differences in the genetic backgrounds of Iranian patients in case of presenting the cancer (9-11).

Findings of fingolide first-dose effects in the present study were similar to those reported in previous trials. The observed transient HR decrease, which was resolved during the 6-hour monitoring, was similar to the findings of previous trials. The mentioned finding revealed the pharmacological effects of fingolide (9-11). Bradycardia was reported in 2.85% of patients in the present study. The mentioned finding was higher than the value observed in the previous trials (2.1%, 1%, and 0.2%, respectively) (9-11).

The present study reported five (2.05%) cases of cardiac events including first-degree AVB (0.82%) leading to the extension of monitoring period to 24 hours and cardiac symptoms resulting in CCU admission. The presented finding was in contrast with the findings provided by previous studies (0.5%, 5%, and 0.5%, respectively). Furthermore, only one of the mentioned cardiac events was typically asymptomatic in the patients of the present study (9-11). Furthermore, over the one-year follow-up period, one (0.32%) of the patients showed PAC as the cardiac emergency about 6 months after starting fingolide. The mentioned observation led to the discontinuation of fingolide treatment. The mentioned report can be considered as a novel finding as compared with the findings presented in the previous trials. Long-term follow-up of fingolide seems to be necessary for better assessment of the mentioned types of effects.

In the present study, herpes infection was observed as chicken pox and zona in 1.29% of patients. The presented value was less than that of previous trials with different sites of herpes infection involvement (8.7%, 8%, and 2.1%, respectively) (9-11). Reversible ME after discontinuation of treatment was recorded in 0.32% of patients in this study. The presented finding was in line with those of previous trials (0%, < 0.5%, and 0.5%, respectively) (9-11).

The current study suggested an increased risk of seizure using fingolide, which was a different finding in comparison with results of previous trials (0%, 1%, and not reported, respectively) (9-11). This event may be related to the positive family history of seizure in our patients or differences in MRI findings based on the location of the plaques or interaction between MS and fingolimod function (41). The mentioned finding may even be incidental in the present study. Possibly, further studies can reveal whether the drug itself can trigger the onset of this event or not. The higher rate of findings regarding AEs in our study (99.21%) in comparison with that of the previous studies (94.4%, 98%, and 86.0% respectively) may be related to physicians’ and patients’ higher levels of awareness with respect to treatment complications (9-11). As already mentioned, AEs such as AUB, diminished libido, increased appetite can be considered as peculiar findings obtained in the present study. 

A 0.3±0.07-point improvement was obtained in the mean EDSS score, which was indicative of disease stability as compared to the baseline EDSS score before starting fingolide treatment (p<0.001). This level of improvement in our study was higher than that of the previous studies (0.00±0.88, 0.046±1.02, and 0.08±0.79 respectively) (9-11). Patients with a 12-month period of fingolide treatment experienced sustained ARR and disease progression in the present study (p<0.001). Mean ARR at the end of our study was 0.005±0.016, which was significantly lower than that of the previous studies (0.18, 0.21, and 0.16 respectively) (9-11).

Recently, another prospective observational study that used fingolide has been conducted over a 12-month of follow-up on 133 western Iranian RRMS patients with a mean age of 32.55±6.78, a mean EDSS score of 3.3±1.11, a mean time of MS diagnosis 7±3.45 years, and a mean ARR of 1.8±1.35. As compared with our patients, the mentioned trial had a smaller number of patients with an older age range, a longer duration of disease, and a higher EDSS score and ARR. This study reported AEs such as UTI (13.33%), URI (3.33%), hypertension (0%), headache (16.66%), and cardiac events (35%). All of the reported findings in this study were similar to the results of our study. Twelve months after the intervention, the mean ARR and EDSS scores were changed to 0.27±0.58 and 2.97±1.17, respectively. The mentioned mean scores indicated a significant decrease as compared to the baseline mean scores (both P=0.001) and were similar to the findings of our study. 23.33% of the patients suffered from at least one relapse and 76.66% of the patients were relapse-free. Both of the mentioned percentages were lower than those of our study (17.5% and 82.5%, respectively). Overall, the reported cases of AEs were 66.66%, which was higher than that of our finding (99.21%). Moreover, only one patient suffered from SAEs, which led to fingolide discontinuation. The mentioned value was lower than that of our study (8.33%) (36). Another randomized trial that evaluated fingolide versus high-dose interferon beta-1a in Iranian RRMS patients was conducted on 120 patients with a mean age of 38.22±9.09, a mean EDSS score of 2.43±1.44, a mean MS diagnosis time of 5.80±5 years, and a mean replase rate of 2.27±0.92. As compared with our study, this trial had a smaller number of patients with an older age range, a shorter duration of disease, and a higher EDSS score. Eighteen months after the intervention, the mean relapse rate changed to 0.6±0.55, which was significantly lower than that of the high-dose interferon group (P=0.002). This finding was inconsistent with the findings of our study (p<0.001) (37). This study had its own limitations. The first limitation was lack of control group, blinding, and limited follow-up time, which might have affected the results. The important limitation of our study was lack of making comparisons between fingolide and the brand fingolimod because of economic issues and sanction effects. Score improved and attack numbers reduced after using fingolide. Moreover, the recorded adverse events (AEs) and serious adverse events (SAEs) were acceptable in this study as compared with previous studies. The obtained findings suggest that fingolide^R^ 0.5 mg (Fingolimod, osvahpharma, Tehran, Iran) is safe, and its safety profile does not significantly differ from that of the branded fingolimod reported in previous studies.

## Abbreviations:

Adverse Events (AEs) , Absolute Lymphocyte Count (ALC), Acute Lymphoblastic Leukemia (ALL), Alanine Transaminase (ALT), Annualized Relapse Rate (ARR), Aspartate Transaminase (AST), Atrioventricular Blocks (AVB_s_), Beta-Human Chorionic Gonadotropin (β-HCG), Complete Blood Count with Differential (CBC/diff), Coronary Care Unit (CCU), Central Nervous System (CNS), Cerebrovascular Accident (CVA), Disease Modifying Drugs (DMD_s_), Electrocardiography (ECG), Expanded Disability Status Scale (EDSS), Glatiramer Acetate (GA)

Heart Rate (HR), Iranian Registry of Clinical Trial (IRCT), Liver Functional Tests (LFTs), Myocardial Infarction (MI), Millimeter Hg or millimeter of mercury (mm Hg), Magnetic Resonance Imaging (MRI), Multiple Sclerosis (MS), Optical Coherence Tomography (OCT), Premature Atrial Contraction (PAC), Percentage of Brain Volume Change (PBVC), Progressive Multifocal Leukoencephalopathy (PML), Personal Protective Division (PPD), Reversible Cerebral Vasoconstriction Syndrome (RCVS), Respiratory Rate (RR), Relapsing-Remitting Multiple Sclerosis (RRMS), Serious Adverse Events (SAEs), Sphingosine-1-Phosphate (S1P), Statistical Package for the Social Sciences (SPSS), Transient Ischemic Attack (TIA), Urine Analysis (U/A), Urinary Tract Infection (UTI), Upper Respiratory Tract Infection (URI), Varicella-Zoster Virus (VZV)
